# Supplementary material for: Polymorphisms in Pfkelch13 domains before and after the introduction of artemisinin-based combination therapy in Southwest Nigeria
Source: PLoS One. 2025 Mar 31;20(3):e0316479. doi: 10.1371/journal.pone.0316479 (PMC11957316; doi:10.1371/journal.pone.0316479)

Samples: 12435
Bases: 833
Average spacing: 15

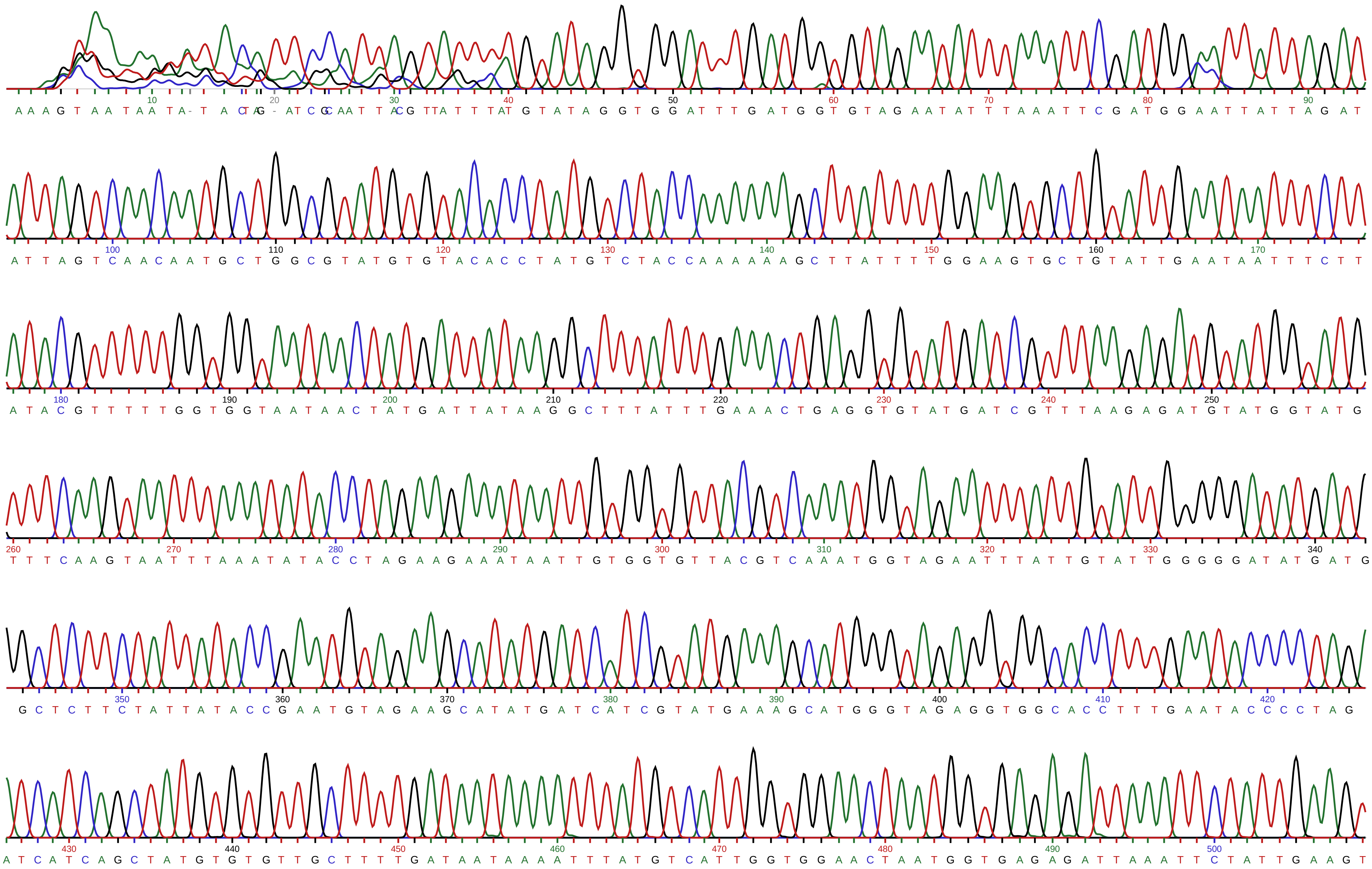

Samples: 12435
Bases: 833
Average spacing: 15

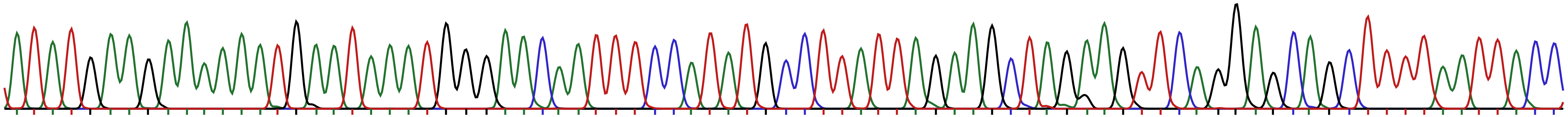

510 520 530 540 550 560 570 580 590

ATATGAAGAAAAAATGAATAAATGGGAACAATTTCCATATGCCTTATTAGAAGCTAGAAGTTCAGGAGCAGCTTTTAATTACC

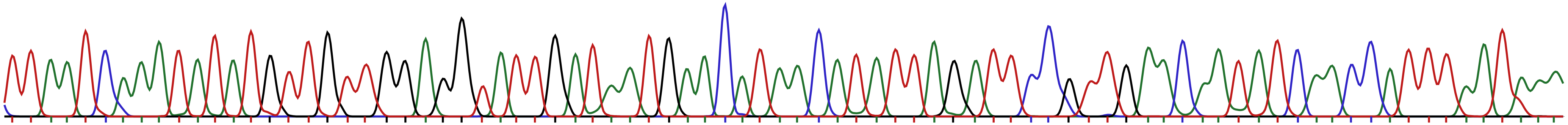

600 610 620 630 640 650 660 670

TTAATCAAATATATGTTGTTGGAGGTATTGATAATGAACATAACATATTAGATTCCGTTGAACAATATCAACCATTTAATAAA

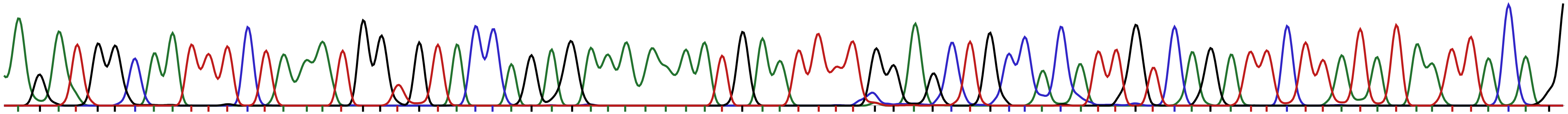

680 690 700 710 720 730 740 750

AGATGGCAATTTCTAAATGGTGTACCAGAGAAAAAAATGAATTTTGGAGCTGCCACATTGTCAGATTCTTATATAATTACAG

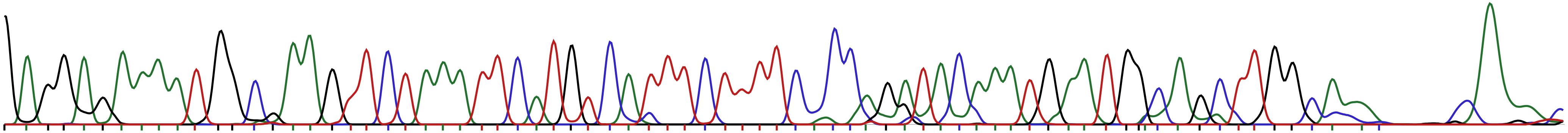

760 770 780 790 800 810 820 830

GAGGAGAAAATGGCGAAGTTCTAAATTCATGTCATTTCTTTTCACCAGATACAAATGAATGGACAGCTTGGCAAC

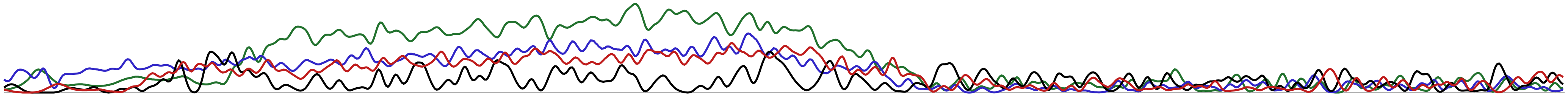

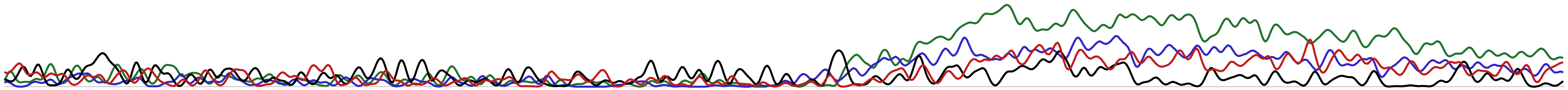

Samples: 12435
Bases: 833
Average spacing: 15

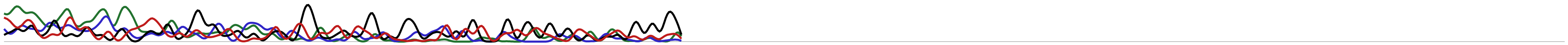

Supplement: Supporting information 2 — (ZIP) [file pone.0316479.s002.zip › 064KNIFW_PREMIX_Plate_CORKELCH_G12.pdf]
